# Trends in Hospitalizations for Peptic Ulcer Disease, United States, 1998–2005^1^

**DOI:** 10.3201/eid1609.091126

**Published:** 2010-09

**Authors:** Lydia B. Feinstein, Robert C. Holman, Krista L. Yorita Christensen, Claudia A. Steiner, David L. Swerdlow

**Affiliations:** Author affiliations: Centers for Disease Control and Prevention, Atlanta, Georgia, USA (L.B. Feinstein, R.C. Holman, K.L. Yorita Christensen, D.L. Swerdlow);; Agency for Healthcare Research and Quality, Rockville, Maryland, USA (C.A. Steiner)

**Keywords:** peptic ulcer, duodenal ulcer, gastric ulcer, gastrojejunal ulcer, Helicobacter pylori, hospitalizations, enteric infections, bacteria, research

## Abstract

Infection with *Helicobacter pylori* increases the risk for peptic ulcer disease (PUD) and its complications. To determine whether hospitalization rates for PUD have declined since antimicrobial drugs to eradicate *H. pylori* became available, we examined 1998–2005 hospitalization records (using the Nationwide Inpatient Sample) in which the primary discharge diagnosis was PUD. Hospitalizations for which the diagnosis was *H. pylori* infection were also considered. The age-adjusted hospitalization rate for PUD decreased 21% from 71.1/100,000 population (95% confidence interval [CI] 68.9–73.4) in 1998 to 56.5/100,000 in 2005 (95% CI 54.6–58.3). The hospitalization rate for PUD was highest for adults >65 years of age and was higher for men than for women. The age-adjusted rate was lowest for whites and declined for all racial/ethnic groups, except Hispanics. The age-adjusted *H. pylori* hospitalization rate also decreased. The decrease in PUD hospitalization rates suggests that the incidence of complications caused by *H. pylori* infection has declined.

## Abstract 2

TOC summary: Decreased hospitalization rates suggest decline in complications from *Helicobacter pylori* infection.

Peptic ulcer disease (PUD) is a common illness that affects >6 million persons in the United States each year, causing considerable illness and a large economic cost to the healthcare system (*1*). Infection with *Helicobacter pylori* substantially increases the risk for PUD and its complications (*2*). Appropriate antimicrobial drug regimens to eradicate the infection and cure ulcers have been available since Marshall and Warren discovered *H. pylori* as an etiologic agent of ulcers in the early 1980s (*3*). Eradicating these infections prevents recurrence and ulcer complications such as bleeding or perforation (*4**–**6*). Therefore, a decline in hospitalizations for PUD and its complications could be expected since treatment for *H. pylori* infection became available.

Although rates of hospitalization for PUD declined in the United States during the 1980s and 1990s, rates remained high (*7*,*8*). One reason was the lack of knowledge among the general public and clinicians about the link between *H. pylori* and PUD (*9**–**11*). The Centers for Disease Control and Prevention, in collaboration with partners from other federal agencies, academic institutions, and private industry, initiated an educational campaign in 1997 to increase awareness of the relationship (*9*). The goals of the campaign were to promote the increased use of appropriate antimicrobial drug treatment to eradicate *H. pylori,* which would thus lead to a further decline in rates of hospitalization for PUD and its complications. Accordingly, reducing hospitalizations for PUD 35% from the 1998 baseline rate of 71/100,000 population to 46/100,000 population by the year 2010 was included in the Healthy People 2010 objectives that were developed in 1998 by the US Department of Health and Human Services (*12*).

The prevalence of *H. pylori* infections and their associated conditions can vary considerably among population groups within the same country. Racial and ethnic differences have been noted, with blacks more affected than whites and Mexican-Americans more affected than non-Hispanic whites and non-Hispanic blacks (*12**,**13*). A recent meta-analysis in which researchers adjusted for age and socioeconomic status, showed that *H. pylori* infection was significantly associated with male sex in 18 adult populations (*14*). In addition, male patients were hospitalized more often for duodenal ulcers than were female patients (*15**,**16*). The prevalence of *H. pylori* infection and PUD can also vary by geographic location, socioeconomic status, and age (*13**,**17*).

Although recent studies have suggested that rates of PUD have declined in European countries and in non-European countries outside the United States (*16**,**18**–**20*), the overall recent national trends of PUD in the United States have not been described. To determine whether rates of hospitalization due to PUD and its complications have decreased and to describe the demographic characteristics of hospitalized persons with PUD, we conducted a retrospective analysis of hospital discharge data for PUD in the United States from 1998 through 2005.

## Methods

Using the Nationwide Inpatient Sample (NIS) (*21*), we analyzed hospital discharge data during 1998–2005 for the general US population. The NIS is the largest all-payer, inpatient-care database in the United States; it is produced by the Healthcare Cost and Utilization Project (HCUP), sponsored by the Agency for Healthcare Research and Quality in partnership with public and private statewide data organizations (*21**,**22*). The NIS is a stratified probability sample of hospitals in participating states designed to approximate a 20% sample of all US community hospitals. Hospitals in the sampling frame include short-term, nonfederal general and specialty hospitals from as many as 37 states (in 2005). We calculated national hospitalization estimates using discharge weights developed by HCUP.

Diagnosis codes from the International Classification of Diseases, 9th Revision, Clinical Modification (ICD-9-CM), were used to define PUD in the following terms: peptic ulcer (533), gastric ulcer (531), gastrojejunal ulcer (534), and duodenal ulcer (532) (*23**)*. Since the first-listed diagnosis is the condition chiefly responsible for the hospitalization, hospitalizations in which 1 of these codes was listed as the first diagnosis were considered to be more specific than hospitalizations in which 1 of these codes was listed as 1 of as many as 15 diagnoses. We limited the analysis to rates of hospitalization for first-listed PUD diagnoses, unless otherwise stated. In a separate analysis, we selected records with *H. pylori* infection as 1 of as many as 15 diagnoses (ICD-9-CM code 041.86), without regard to PUD on the record.

The PUD and *H. pylori* hospitalizations were examined by age group (<20, 20–44, 45–64, and >65 years of age), sex, race/ethnicity (white, black, Hispanic, and Asian or Pacific Islander), geographic region (Northeast, Midwest, South, and West), designated ulcer type (peptic, gastric, gastrojejunal, and duodenal), other diagnoses listed along a PUD diagnosis, and procedures. Race/ethnicity was missing in the record for 26.0% of hospitalizations.

We selected first-listed hospitalizations for gastritis/duodenitis (ICD-9-CM code 535) as a comparison group to ensure that a change in the PUD hospitalization rate was not attributable to changes in diagnoses resulting from the increased specificity associated with endoscopy. Hospitalizations for all diagnoses were also examined as a comparison group to ensure that a change in hospitalizations for PUD was not merely a reflection of a change in the total number of hospitalizations for all diagnoses.

Annual average hospitalization rates were expressed as the number of hospitalizations per 100,000 population. The hospitalization rates were calculated by using the weighted number of hospitalizations and the census population for each year of the study period from HCUP (*24**,**25*). SEs for the hospitalization estimates were calculated by using SUDAAN software and discharge weights provided by HCUP to account for the sampling design; SEs were used to calculate 95% confidence intervals (CIs) for the rates (*25*). If the relative SE of national estimates exceeded 0.30, or if the number of unweighted outpatient visits or hospitalizations in a strata was <30, the estimates were considered unreliable and were not shown here (*26*).

A weighted least-squares technique was used to assess a linear trend in the annual hospitalization rate for ulcer types during the 8-year study period of 1998–2005; the independent variable was year and the dependent variable was the rate. A modification to the classical regression technique was necessary to account for the changing NIS survey design over the study period, as described by Gillum et al. (*27*). In this method, a regression line is fit to the data, and the resulting slope is tested for difference from zero by using a Wald test for significance; autocorrelation was not assessed. A p value <0.05 was considered significant in this study.

Age-adjusted rates were calculated by using the direct method, taking into account the survey design, and using the projected 2000 US Census population as the reference population (*28*). In the direct method, the age-adjusted rate represents what the rate would be if the study population had the same age distribution as a reference population (i.e., the projected US Census 2000 age distribution). This method is used to remove confounding by age when rates are compared over time or across populations with different age distributions. To calculate age-adjusted rates, the age group–specific rates (hospitalizations/population) were multiplied by weights representing the proportion of the reference population belonging in the corresponding age group; the resulting quantities were summed to obtain the age-adjusted rate (*28*). We report the age-adjusted PUD hospitalization rates; the overall age-adjusted rate did not differ from the unadjusted rate, although the rates for some groups did differ.

## Results

### Overall PUD Hospitalization Rates

A total of 1,453,892 first-listed PUD hospitalizations were estimated for 1998–2005, with an average annual age-adjusted hospitalization rate of 63.6/100,000 population (95% CI 62.9–64.3) (Table 1). The hospitalization rate was highest for adults >65 years of age (299.8/100,000 population) and decreased with decreasing age group. Overall, age-adjusted hospitalization rates were significantly higher for male patients than for female patients (71.9/100,000 population [95% CI 71.0–72.7] and 56.3/100,000 population [95% CI 55.6–57.0], respectively). The rates were significantly higher for male patients of all age groups and of all race/ethnicity groups.

**Table 1:** Number of hospitalizations and age-adjusted and age-specific rates of hospitalization for first-listed discharge diagnoses of peptic ulcer disease, overall and by sex, United States, 1998–2005*

Characteristic	Male patients			Female patients			Overall	
No. (SE)	Rate† (95% CI)	No. (SE)	Rate† (95% CI)	No. (SE)	Rate† (95% CI)
Age, y								
<20	7,851 (320)	2.4 (2.2–2.6)		4,926 (349)	1.6 (1.4–1.8)		12,803 (602)	2.0 (1.8–2.2)
20–44	113,742 (1,344)	27.0 (26.4–27.6)		72,638 (945)	17.5 (17.1–18.0)		186,557 (1,979)	22.3 (21.8–22.8)
45–64	236,381 (2,206)	92.6 (90.9–94.3)		165,013 (1,628)	61.3 (60.2–62.5)		401,581 (3,480)	76.6 (75.3–77.9)
>65	380,016 (3,555)	322.5 (316.6–328.4)		472,590 (4,224)	283.6 (278.6–288.6)		852,720 (7,409)	299.8 (294.7–304.9)
Race/ethnicity‡								
White	392,199 (5,508)	48.7 (47.7–49.6)		408,120 (5,712)	40.4 (39.5–41.2)		800,358 (10,974)	44.2 (43.3–45.0)
Black	70,453 (1,968)	69.7 (67.1–72.3)		59,045 (1,733)	46.4 (44.5–48.2)		129,499 (3,577)	56.8 (54.7–58.8)
Hispanic	48,076 (1,661)	58.4 (55.1–61.7)		33,179 (1,324)	38.7 (36.1–41.2)		81,270 (2,887)	48.0 (45.3–50.8)
Asian/Pacific Islander	23,268 (1,158)	68.2 (63.0–73.5)		14,789 (767)	38.1 (34.7–41.6)		38,056 (1,846)	51.8 (47.8–55.8)
Ulcer type								
Gastric	343,079 (3,011)	33.5 (33.1–34.0)		427,469 (3,750)	33.6 (33.1–34.0)		770,785 (6,347)	33.7 (33.3–34.2)
Peptic	40,009 (678)	3.8 (3.7–3.9)		51,452 (818)	4.1 (4.0–4.2)		91,524 (1,313)	4.0 (3.9–4.1)
Duodenal	340,161 2,973	33.1 (32.9–33.5)		217,946 (2,055)	17.1 (16.8–17.4)		558,443 (4,676)	24.4 (24.1–24.7)
Gastrojejunal	14,772 (335)	1.4 (1.4–1.5)		18,336 (504)	1.5 (1.4–1.6)		33,142 (699)	1.4 (1.4–1.5)
Total	738,020 (5,897)	71.9 (71.0–72.7)		715,203 (5,824)	56.3 (55.6–57.0)		1,453,892 (11,201)	63.6 (62.9–64.3)

*Diagnosis codes 531–534 from the International Classification of Diseases, 9th Revision, Clinical Modification. National estimates determined by using the Nationwide Inpatient Sample (21). †Per 100,000 population. ‡Race/ethnicity was missing for 26.0% of hospitalized patients. Data were insufficient for the race/ethnicity category of American Indian/Alaska Native.

Overall, age-adjusted hospitalization rates were significantly lower for whites (44.2/100,000 population; 95% CI 43.3–45.0) than for each of the other racial/ethnic groups (Table 1). This rate difference for male patients was similar across groups of various races and ethnicities. Among female patients, the rate was significantly higher for blacks than those for each of the other racial/ethnic groups.

The average annual age-adjusted hospitalization rate was higher for patients with ulcers designated gastric (33.7/100,000 population; 95% CI 33.3–34.2) than for all patients with other ulcer designations (Table 1). However, a gender-specific comparison showed that for male patients, the hospitalization rate for ulcers designated gastric was comparable to that for ulcers designated duodenal (33.5/100,000 population [95% CI 33.1–34.0] and 33.1/100,000 population [95% CI 32.9–33.5]). Among female patients, the hospitalization rate for ulcers designated gastric (33.6/100,000 population; 95% CI 33.1–34.0) was almost double that for ulcers designated duodenal (17.1/100,000 population; 95% CI 16.8–17.4).

### Trends over Time in PUD Hospitalization Rates

**Figure 1:**
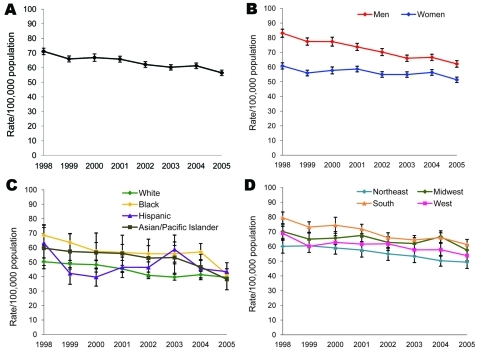
Age-adjusted hospitalization rates for first-listed discharge diagnoses of peptic ulcer disease (diagnosis codes 531–534 from the International Classification of Diseases, 9th Revision, Clinical Modification), United States, 1998–2005. A) Overall age-adjusted hospitalization rate. B) Age-adjusted hospitalization rate by gender. C) Age-adjusted hospitalization rate by race/ethnicity. D) Age-adjusted hospitalization rate by region. Source: Nationwide Inpatient Sample (*21*). Race/ethnicity information was missing for 26.0% of hospitalizations.

The overall age-adjusted hospitalization rate for PUD decreased 21%, from 71.1/100,000 population (95% CI 68.9–73.4) in 1998 to 56.5/100,000 population (95% CI 54.6–58.3) in 2005 (Table 2; Figure 1). The hospitalization rate appeared to decline for all age groups (19%–22%), except children <20 years of age, for whom no significant change occurred during the study period. Although the hospitalization rate was higher for male patients than for female patients in 1998 (83.1/100,000 and 60.8/100,000 population, respectively) and in 2005 (62.2/100,000 and 51.3/100,000 population, respectively), the difference decreased because of a greater decline for male patients (25%) than for female patients (16%). The hospitalization rate was significantly lower in 2005 than in 1998 for all racial/ethnic groups, except for Hispanics; the greatest decline was found for blacks (40%) and the least decline was observed for whites (22%). The hospitalization rate was also significantly lower in 2005 than in 1998 in all regions.

**Table 2:** Number of hospitalizations and the age-adjusted and age-specific hospitalization rates for first-listed discharge diagnoses of peptic ulcer disease, United States, 1998–2005*

Characteristic	1998			2005		% Rate change	β-coefficient‡
	No. (SE)	Rate† (95% CI)		No. (SE)	Rate† (95% CI)		
Age, y							
<20	1,524 (133)	1.9 (1.6–2.2)		1,924 (290)	2.4 (1.7–3.0)	+26	0.016§
20–44	26,420 (817)	25.5 (23.9–27.0)		21,523 (656)	20.5 (19.3–21.7)	−20	−0.648¶
45–64	50,446 (1,164)	86.6 (82.7–90.5)		50,993 (1,326)	70.0 (66.4–73.6)	−19	−1.911¶
≥6>65	115,181 (2,750)	332.7 (317.1–348.3)		95,349 (2,449)	259.2 (246.1–272.2)	−22	−8.759¶
Sex							
M	100,721 (2,159)	83.1 (80.4–85.6)		84,883 (2,013)	62.2 (59.9–64.4)	−25	−2.164¶
F	92,831 (2,080)	60.8 (58.7–62.9)		84,808 (2,040)	51.3 (49.5–53.2)	−16	−0.886¶
Race/ethnicity#							
White	111,092 (4,086)	50.3 (47.7–52.9)		91,939 (3,765)	39.5 (37.3–41.8)	−22	−1.696¶
Black	18,188 (1,428)	68.6 (61.7–75.6)		12,794 (899)	41.4 (37.5–45.4)	−40	−2.093¶
Hispanic	10,850 (1,310)	63.3 (50.7–75.9)		11,079 (1,034)	43.4 (37.3–49.6)	−31	0.308§
Asian/Pacific Islander	4,526 (673)	59.7 (45.4–73.9)		4,220 (520)	38.2 (30.9–45.4)	−36	−1.620**
Region							
Northeast	33,578 (1,746)	60.1 (55.5–64.7)		29,046 (1,740)	49.3 (45.1–53.6)	−18	−1.697¶
Midwest	44,910 (1,909)	70.1 (65.5–74.7)		39,097 (1,562)	57.5 (53.8–61.1)	−18	−0.964**
South	76,773 (2,551)	79.5 (75.6–83.4)		66,969 (2,711)	61.3 (57.8–64.8)	−23	−2.046¶
West	38,315 (1,707)	68.9 (64.2–73.7)		34,746 (1,519)	53.8 (50.2–57.3)	−22	−1.129††
Total	193,576 (4,014)	71.1 (68.9–73.4)		169,858 (3,889)	56.5 (54.6–58.3)	−21	−1.501¶

*Diagnosis codes 531–534 from the International Classification of Diseases, 9th Revision, Clinical Modification. National estimates were determined by using the Nationwide Inpatient Sample (21). †Per 100,000 population. ‡β-coefficient from linear regression analysis, using a weighted least-squares technique, of the annual rate over the years of the study period. The p value corresponds to the test of the null hypothesis that the coefficient for year is zero. § p>0.05. ¶p<0.001. #Race/ethnicity was missing for 26.0% of the hospitalizations. Data were insufficient for the race/ethnicity category American Indian/Alaska Native. **0.01<p<0.05. ††0.001<p<0.01.

The age-adjusted rate for hospitalizations for gastritis/duodenitis (selected as a comparison group to ensure that a change in the PUD hospitalization rate was not attributable to changes in diagnosis coding practices) decreased 16%, from 55.0/100,000 population (95% CI 52.6–57.4) in 1998 to 46.0/100,000 population (95% CI 44.4–47.7) in 2005. The rate of hospitalization for all diagnoses (included as a comparison group to ensure that a change in hospitalizations for PUD was not merely a reflection of a change in the total number of hospitalizations for all diagnoses) did not change significantly during the study period.

### Procedures and Other Listed Diagnoses

**Figure 2:**
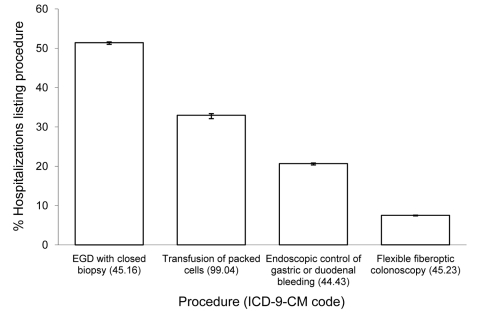
Hospital procedures most frequently listed with first-listed discharge diagnoses of peptic ulcer disease (diagnosis codes 531–534 from the International Classification of Diseases, 9th Revision, Clinical Modification [ICD-9-CM]), United States, 1998–2005. Source: Nationwide Inpatient Sample (*21*). EGD, esophago-gastroduodenoscopy.

**Figure 3:**
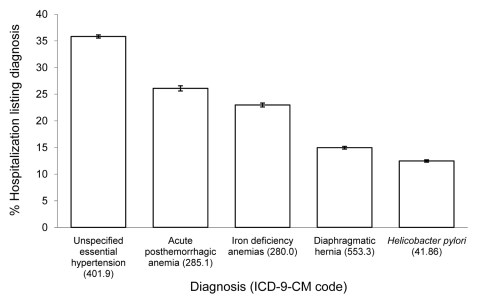
Other diagnoses most frequently listed with first-listed discharge diagnoses of peptic ulcer disease (PUD) (diagnosis codes 531–534 from the International Classification of Diseases, 9th Revision, Clinical Modification [ICD-9-CM]), United States, 1998–2005. Source: Nationwide Inpatient Sample (*21*). Iron deficiency anemias, iron deficiency anemias secondary to blood loss**.**

**Figure 4:**
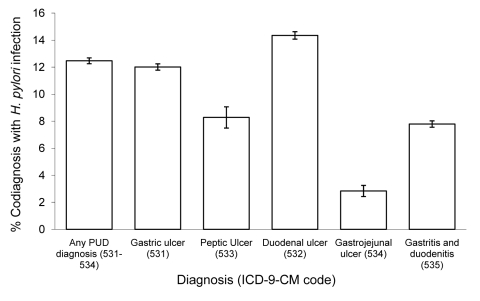
Proportion of first-listed ulcer diagnoses with a co-diagnosis of *Helicobacter pylori* infection (diagnosis codes 531–534 from the International Classification of Diseases, 9th Revision, Clinical Modification [ICD-9-CM]), by ulcer type, United States, 1998–2005. Source: Nationwide Inpatient Sample (*21*). PUD, peptic ulcer disease.

Esophago-gastroduodenoscopy (EGD) with closed biopsy of ≥1 sites involving the esophagus, stomach, or duodenum was the most common procedure performed in patients with PUD listed as first reason for hospitalization (Figure 2). Transfusion of packed red blood cells, endoscopic control of gastric or duodenal bleeding and flexible fiberoptic colonoscopy were also common. Many diagnoses frequently were listed with PUD hospitalizations. Among these, unspecified essential hypertension, acute posthemorrhagic anemia, iron deficiency anemia secondary to blood loss, diaphragmatic hernia, and *H. pylori* infection were the most common (Figure 3). A greater proportion of ulcers designated duodenal were listed with an *H. pylori* co-diagnosis than any other ulcer designation considered (Figure 4).

### Hospitalization Rates for *H. pylori* Infections

The overall age-adjusted rate of hospitalization that included any discharge diagnosis of *H. pylori* infection decreased 47%, from 35.9/100,000 population (95% CI 34.3–37.5) in 1998 to 19.2/100,000 population (95% CI 18.3–20.1) in 2005 (Table 3). The hospitalization rate increased with age and declined during 1998–2005 for all age groups except children <20 years of age. The greatest percentage rate decrease was observed for adults >65 years of age, for whom the hospitalization rate decreased 54%, from 163.5/100,000 population (95% CI 152.9–174.0) in 1998 to 75.4/100,000 population (95% CI 70.0–80.8) in 2005. The hospitalization rate for male patients was only slightly higher than that for female patients, and the decline in rates was similar for both groups. In 1998, the hospitalization rate was higher for blacks (44.1/100,000 population; 95% CI 39.7–48.6) and Hispanics (41.8/100,000 population; 95% CI 34.0–49.6) than for whites (23.2/100,000 population; 95% CI. 21.7–27.9) and Asian/Pacific Islanders (34.0/100,000 population; 95% CI 26.2–41.9). In 2005, the same pattern was observed; hospitalization rate was significantly higher for blacks (23.6/100,000 population; 95% CI 21.1–26.1) and Hispanics (24.5/100,000 population; 95% CI 21.1–27.9) than for whites (10.3/100,000 population; 95% CI 9.6–11.0) and Asian/Pacific Islanders (15.8/100,000 population; 95% CI 12.0–19.6). Rates declined significantly for all racial/ethnic groups, except for Hispanics; the greatest percentage rate decrease was observed for whites (56%). The hospitalization rates were significantly different in 1998 and 2005 in all geographic regions. In 1998, the hospitalization rate was higher in the South (39.3/100,000 population; 95% CI 36.4–42.1) than in the West and Northeast regions (33.6/100,000 and 31.4/100,000 population, respectively; 95% CI 30.8–36.3 and 27.8–35.1, respectively). By 2005, the hospitalization rate was higher in the South (21.6/100,000 population; 95% CI 19.8–23.4) and in the Northeast (19.3/100,000 population; 95% CI. 17.6–21.0) than in the West (16.5/100,000 population; 95% CI 14.8–18.1) because the decline apparently occurred more slowly in the Northeast (39%) than in all other regions.

**Table 3:** Number of hospitalizations and the age-adjusted and age-specific hospitalization rates for any-listed discharge diagnoses that included *Helicobacter pylori* infection, United States, 1998–2005*

Characteristic	1998			2005		% Rate change	β-coefficient‡
	No. (SE)	Rate† (95% CI)		No. (SE)	Rate† (95% CI)		
Age, y							
<20	1,320 (151)	1.7 (1.3–2.0)		1,718 (276)	2.1 (1.4–2.8)	+24	0.040§
20–44	14,617 (648)	14.1 (12.9–15.3)		10,391 (457)	9.9 (9.0–10.7)	−30	−0.436¶
45–64	25,287 (955)	43.4 (40.2–46.6)		17,923 (724)	24.6 (22.7–26.6)	−43	−2.265¶
>65	56,593 (1,866)	163.5 (152.9–174.0)		27,733 (1,009)	75.4 (70.0–80.8)	−54	−10.647¶
Sex							
M	47,162 (1,450)	38.9 (37.2–40.6)		28,535 (946)	20.6 (19.6–21.6)	−47	−1.854¶
F	50,656 (1,830)	33.4 (31.7–35.0)		29,220 (1,078)	18.0 (17.0–18.9)	−46	−1.909¶
Race/ethnicity#							
White	51,235 (2,353)	23.2 (21.7–24.7)		23,735 (1,117)	10.3 (9.6–11.0)	−56	−1.649¶
Black	11,724 (887)	44.1 (39.7–48.6)		7,480 (561)	23.6 (21.1–26.1)	−47	−1.926¶
Hispanic	7,305 (839)	41.8 (34.0–49.6)		7,075 (720)	24.5 (21.1–27.9)	−41	0.131§
Asian/Pacific Islander	2,563 (361)	34.0 (26.2–41.9)		1,771 (252)	15.8 (12.0–19.6)	−54	−1.471¶
Region							
Northeast	17,551 (1,588)	31.4 (27.8–35.1)		11,285 (681)	19.3 (17.6–21.0)	−39	−1.392¶
Midwest	23,644 (1,430)	36.9 (33.6–40.2)		12,116 (722)	17.9 (16.3–19.5)	−52	−2.229¶
South	37,929 (2,100)	39.3 (36.4–42.1)		23,603 (1,476)	21.6 (19.8–23.4)	−45	−2.193¶
West	18,699 (1,038)	33.6 (30.8–36.3)		10,766 (738)	16.5 (14.8–18.1)	−51	−1.493¶
Total	97,823 (3,156)	35.9 (34.3–37.5)		57,770 (1,925)	19.2 (18.3–20.1)	−47	−1.884¶

*Diagnosis codes 41.86 from the International Classification of Diseases, 9th Revision, Clinical Modification. National estimates were determined by using the Nationwide Inpatient Sample (*21*). All *H. pylori* diagnoses are 1 of as many as 15 diagnoses. †Per 100,000 population. ‡β-coefficient from linear regression analysis, using a weighted least-squares technique, of the annual rate over the years of the study period. The p value corresponds to the test of the null hypothesis that the coefficient for year is zero. §p≥0.05. ¶p<0.001. #Race/ethnicity was missing for 26.0% of the patients hospitalized. Data were insufficient for the race/ethnicity category American Indian/Alaska Native.

## Discussion

Our analysis of hospital discharge records from a nationally representative sample of US hospitals indicates that the overall age-adjusted rate of hospitalization for PUD declined during 1998–2005. This finding is consistent with decreases previously observed for PUD hospitalizations in the United States in several studies from the 1970s through the 1990s (*7**,**8**,**14**,**17**,**29**)*. One study by Manuel et al. did not observe a downward trend in PUD hospitalizations during 1996–2005; however, that study included only 5 hospitals (*30*). We analyzed data for first-listed PUD hospitalizations to limit data to hospitalizations for care specifically for ulcer-related issues. In addition, >50% of patients in our study had an EGD with closed biopsy of ≥1 sites involving the esophagus, stomach, or duodenum (Figure 2). EGD with biopsy is a reliable technique to differentiate between PUD and other causes of abdominal pain such as gastritis (*17*). Thus, it appears that the data in this study are reflective of patients who were truly hospitalized primarily for PUD or its complications.

Decreases in PUD hospitalizations are likely attributable to an underlying decline in *H. pylori* prevalence (*7**,**15*). However, declines in PUD hospitalizations have also been attributed to changes in diagnosis coding because of improved diagnostic specificity associated with endoscopy (*31*). If the decrease in PUD hospitalizations was attributable to changes in diagnosis coding, the decrease in PUD hospitalizations would be inversely related to a rise in hospitalizations for gastritis/duodenitis. We found that hospitalization rates declined for gastritis/duodenitis and for PUD, which indicates that the results cannot be attributable to changes in diagnosis coding practices. Furthermore, because the hospitalization rate for all diagnoses did not change significantly from 1998 to 2005, the decline in the PUD hospitalization rate observed would not merely reflect general trends in hospitalization. The overall rate for any listed *H. pylori* diagnosis declined significantly during the study period, which suggests that a decrease in rates of *H. pylori* infections may be partially responsible for the decrease in hospitalizations for PUD.

In our study, the overall rate of hospitalizations for PUD differed according to the patient’s age, sex, race/ethnicity, and region. The highest rates of hospitalization for those with both PUD and *H. pylori* infection were for adults >65 years of age and decreased with each subsequent age group. This finding may result from an underlying birth cohort effect, in this case a decrease in *H. pylori* incidence for younger generations because of improved sanitation and fewer risk factors for transmission (*18**,**32**,**33*). A similar percentage change in rate of PUD hospitalizations was observed for all age groups >20 years. The comparable declines for these age groups may be partially attributable to increased use of *H. pylori* eradication therapy during 1998–2005, perhaps because of increased awareness among clinicians and patients of the association between *H. pylori* and PUD (*9*).

In this study, the overall rate of hospitalization for PUD in 1998 was higher for male patients than for female patients. However, by 2005 this difference had narrowed considerably because of a greater decrease in rates for male patients than for female patients. A 1985 study that examined data from the National Center for Health Statistics also recognized a trend toward comparable rates of hospitalization for both sexes (*34*). Our study also found differences in hospitalization rates between sexes by designated ulcer type; duodenal ulcer hospitalization rates were higher for male patients than for female patients. This finding is consistent with hospital admission data from the United Kingdom (*15*,*16*).

A study that used a national sample of US hospital discharge records noted differences in the hospitalization rate for PUD between racial/ethnic groups; blacks were more frequently hospitalized for PUD than whites in 1998 (*13*). Although we also found that rate of hospitalization for PUD was higher for blacks than for whites, the rate appears to be declining more rapidly for blacks than for whites. In addition, although rates were significantly lower for whites than for those in other racial/ethnic categories in 1998, by 2005 this rate difference was no longer significant because for whites, the decline apparently occurred more slowly than it did for all other racial/ethnic groups. Differences also varied by sex, as well as race/ethnicity, and suggest that hospitalizations for PUD among nonwhite men may merit further investigation. Race/ethnicity information was missing for patients in 26% of hospitalization records, possibly making comparisons between racial/ethnic groups inaccurate. A study of underreporting of race/ethnicity information in the National Hospital Discharge Survey suggests that hospitals that do not report race/ethnicity information may have a higher proportion of discharges for whites and a lower proportion of discharges for blacks than hospitals that do report race/ethnicity information (*35*). Our study did not examine PUD hospitalizations for American Indians and Alaska Natives because the survey’s sample size was not large enough and did not include visits to Indian Health Service or tribal facilities. However, a previous study showed that among American Indians and Alaska Natives, the prevalence of ulcer-associated conditions was high during 1996–2005, which indicates that hospitalizations for PUD among this group may warrant further study (*36*).

Our study showed similar trends for hospitalizations for PUD and *H. pylori* infection, although we noted some differences. For both PUD and *H. pylori* infections, the rate of hospitalization increased with age, and the age-adjusted hospitalization rate was lower for whites than for persons in any other racial/ethnic group category. In addition, the overall age-adjusted rate of both PUD and *H. pylori* hospitalizations was higher for male patients than for female patients. However, although the age-adjusted PUD hospitalization rate appears to be declining more rapidly among male patients than among female patients, the age-adjusted *H. pylori* infection hospitalization rate appears to be declining at a similar pace for female patients and male patients. The age-adjusted PUD hospitalization rate for Hispanics did not decline significantly, and a decline in the age-adjusted *H. pylori* hospitalization rate for this group was only borderline significant, which suggests that rates among this group may deserve special attention. However, this finding may be biased because of missing information on race/ethnicity.

Our findings in this study show a continued downward trend in the rate of hospitalizations for PUD in the United States. Differences in the rate of decline for PUD hospitalization rates between sexes, racial/ethnic groups, and regions warrant further study. The overall downward trend observed in this study does not seem to be attributable to increases in gastritis/duodenitis hospitalizations or to a decline in total hospitalizations. The decline in the PUD hospitalization rate may be attributable to a birth cohort effect with subsequent declines in *H. pylori* infection prevalence and increased use of successful antibiotic treatments to eradicate *H. pylori* infections. Other factors possibly contributed to the decline in PUD hospitalizations observed in this study, including trends in use of nonsteroidal anti-inflammatory drugs and the availability of over-the-counter H2 antagonists and proton pump inhibitors. Studies on the relationship between PUD hospitalizations and nonsteroidal anti-inflammatory drug use, the possibility of undercoding for *H. pylori* on hospitalization discharge records, and subpopulation analyses would help further guide recommendations and show how to focus interventions. To facilitate further declines in hospitalizations for PUD, patients and clinicians should continue to be educated about the association between *H. pylori* and PUD.
